# HER2 Status in Premalignant, Early, and Advanced Neoplastic Lesions of the Stomach

**DOI:** 10.1155/2015/234851

**Published:** 2015-10-01

**Authors:** A. Ieni, V. Barresi, L. Rigoli, R. A. Caruso, G. Tuccari

**Affiliations:** ^1^Department of Human Pathology “Gaetano Barresi”, Section of Anatomic Pathology, Azienda Ospedaliera Universitaria “Gaetano Martino, University of Messina,” Via Consolare Valeria 1, 98125 Messina, Italy; ^2^Department of Pediatrics, Gynecology and Microbiology Sciences, Azienda Ospedaliera Universitaria “Gaetano Martino, University of Messina,” Via Consolare Valeria 1, 98125 Messina, Italy

## Abstract

*Objectives*. HER2 expression in gastric cancer (GC) has received attention as a potential target for therapy with Trastuzumab. We reviewed the current knowledge on HER2 status in premalignant gastric lesions and in early (EGC) and advanced (AGC) GC to discuss the possible pathogenetic and prognostic roles of HER2 overexpression in GC. *Results*. HER2 overexpression was documented in gastric low-grade (LG) and high-grade intraepithelial neoplasia (HG-IEN), with higher frequency in gastric type dysplasia. HER2 overexpression was significantly associated with disease recurrence and poor prognosis in EGC representing an independent risk factor for lymph node metastases. HER2 overexpression was more frequent in AGC characterized by high grade, advanced stage, and high Ki-67 labeling index. The discordance in HER2 status was evidenced between primitive GC and synchronous or metachronous metastases. *Conclusions*. HER2 overexpression in premalignant gastric lesions suggests its potential involvement in the early steps of gastric carcinogenesis. The assessment of HER2 status in EGC may be helpful for the identification of patients who are at low risk for developing nodal metastases. Finally, the possible discordance in HER2 status between primary GC and its synchronous metastases support routine assessment of HER2 both in the primary GC and in its metastatic lesions.

## 1. Introduction

Although the incidence and mortality from gastric carcinoma (GC) significantly decreased over the last fifty years, this tumor still represents the third most common malignancy and the second leading cause of cancer death worldwide [1]. The high mortality rate from GC is mainly related to late diagnosis and to the lack of programs for early detection of this tumor [2–4]. The EUROCARE-5 results show that the 5-year survival rate to GC is 25.1%, with a significant difference recorded between men and women [1]. Of note, survival with GC varies depending upon the geographic area, with the highest survival rate observed in southern and central Europe and the lowest in Eastern Europe, United Kingdom, and Ireland [1]. Among the European countries, a high incidence of mortality from GC is encountered in Italy [2, 5, 6]; interestingly, a remarkable peculiar geographic variation was reported in this country [2, 5, 6] with the highest death rate in central and northern regions and the lowest in southern Italy [2, 6, 7].

Although the infection from* Helicobacter pylori* (*H. pylori*) is a known trigger of gastric carcinogenesis, many other external and internal events play a role in the development of this neoplasia [8]. Microscopically, GC is preceded by several precancerous lesions, including atrophic gastritis, hyperplasia, intestinal metaplasia, and dysplasia [8–14]. Those conditions are characterized by the accumulation of multiple genetic abnormalities, such as oncogene activation, tumor suppressor gene inactivation, and telomerase reactivation [15, 16], which may originate in part from chromosomal instability (CIN) [16, 17]. The latter consists in the loss or gain of whole chromosomes with aneuploidy and altered DNA copy number or in the partial alteration of chromosomes due to translocation, amplification, or deletion [17, 18]. Hence CIN may lead to the loss or gain of oncogenes, tumor suppressor genes, or genes involved in DNA repair or cell cycle checkpoints [17, 18]. Recently, the Cancer Genome Atlas (TCGA) project classified tumors with CIN as a distinct biomolecular subgroup of GC characterized by the frequent amplification of genes such as HER2, EGFR, MET, FGFR2, and RAS genes (KRAS/NRAS) which are all related to the receptor tyrosine kinase RTK/RAS signaling [19]. In particular, HER2 gene encodes for HER2/erbB2 protein which belongs to the epidermal growth factor receptor family that comprises three other proteins with a similar structure, namely, HER1/erbB1, HER3/erbB3, and HER4/erbB4. HER2 plays an important role in the proliferation and differentiation of normal cells [20] and binding to its ligand gives rise to the creation of homodimers and heterodimers and activation of downstream signaling pathways [20]. Any aberrations in the structure or function of this receptor may lead to uncontrolled cell proliferation, neoplastic development, and progression [20]. Trastuzumab is a humanized monoclonal antibody that selectively targets HER2 receptor and inhibits its downstream signaling pathways in cells with HER2 overexpression [21]. A recent phase III randomized study (ToGA) demonstrated a significant survival benefit in patients affected by advanced GC with HER2 overexpression and treated with combined Trastuzumab and chemotherapy [22]. Hence, in recent years, the evaluation of HER2 overexpression has received attention as a target for novel therapeutic strategies aimed at increasing the survival to GC. In addition, assessment of HER2 status in all GCs at the time of diagnosis has been recommended in order to establish patient eligibility for treatment with Trastuzumab.

In this paper we review the controversial role of HER2 in gastric cancerogenesis and focus on the prevalence and potential prognostic significance of HER2 expression in preneoplastic lesions as well as in early and advanced GC.

## 2. HER2 in Premalignant Gastric Lesions

Although chronic atrophic gastritis and intestinal metaplasia of the stomach are considered to be preneoplastic lesions of GC, some Japanese studies do not clearly indicate a role in gastric carcinogenesis [23, 24]. Therefore dysplasia of the gastric mucosa represents the only universally accepted precancerous lesion of GC. Dysplasia is characterized by a wide range of cellular and structural atypia and it is defined as intraepithelial neoplasia (IEN), a pathological condition which lies between atrophic gastritis and GC [25]. IEN may develop in the gastric epithelium affected or not by intestinal metaplasia and it can be classified into four categories: indefinite for intraepithelial neoplasia, low-grade intraepithelial neoplasia (LG-IEN), high-grade intraepithelial neoplasia (HG-IEN), and suspicious for invasive cancer [26, 27]. The histological distinction between LG and HG IEN relies on the severity of architectural and cytological atypia. In detail, in LG-IEN the mucosa maintains tubular differentiation and the proliferative zone is limited to the outward portion, while in HG-IEN mucosal architecture is distorted and shows crowded irregular glands with marked cellular atypia and diffuse proliferative activity [28]. HG-IEN is associated with increased risk of GC [28–31]. Compared to LG-IEN, it is characterized by higher frequency of genetic abnormalities, including 8q gain, p53 overexpression, e-cadherin loss, and HER2 amplification, which are also present in invasive GC [32–36].

The possible occurrence of HER2 amplification in precancerous lesions was previously investigated in bronchial and breast epithelia [37–40]. HER2 amplification was evidenced in bronchial dysplasia with a role in cellular proliferation, but not in the progression to invasive carcinoma [37, 38]. In addition, HER2 overexpression was documented in breast ductal carcinoma in situ with negative prognostic significance, but not in benign and atypical proliferative lesions [39, 40].

**Figure 1:**
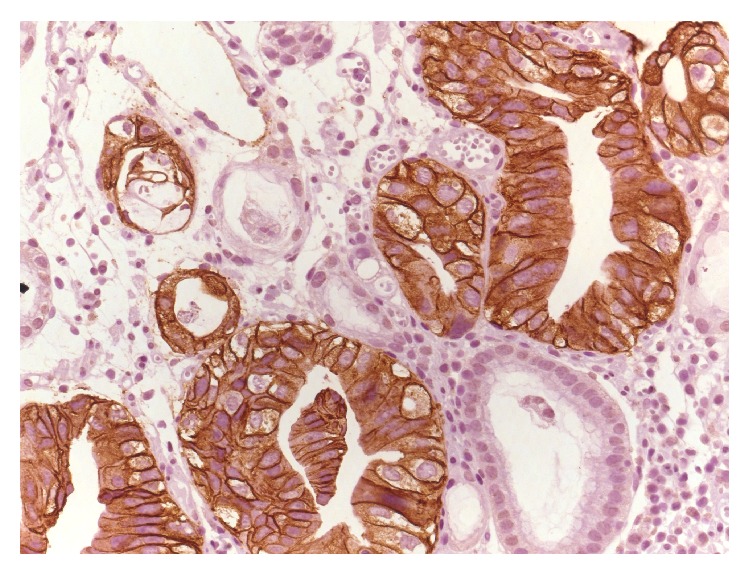
3+ intense HER2 immunoreactivity in gastric HG-IEN. Note absence of staining in normal glands (original magnification, ×400: Mayer's Haemalum nuclear counterstain).

Only few studies investigated HER2 overexpression in gastric dysplasia [36, 41–46]. In a series of surgical and bioptic samples, HER2 immunostaining with 2+/3+ score was evidenced in 12.6% of HG-IEN (Figure 1, authors' collection). Benign gastric mucosa did not show HER2 positivity in any of the specimens, although weak membranous reaction in the foveolae and cytoplasmic staining in specialized glands were observed, as elsewhere previously reported. The comparison of HER2 status between dysplasia and invasive GC showed six cases with concordant 3+ HER2 reactivity and seven with discordant HER2 status; in detail, three cases showed HER2 positivity in the dysplastic epithelium but not in the invasive GC, four cases displayed HER2 overexpression in GC but not in dysplasia [46]. It may be argued that the possible discordant HER2 status between paired dysplasia and GC should indicate that extrapolation of HER2 status of invasive carcinoma based on that observed in dysplasia is not reliable. Moreover, it may pose practical difficulties in assessing HER2 expression in biopsies with high-grade dysplasia transiting to carcinoma, determining false positive results in biopsies, due to the misinterpretation of HER2-positive dysplasia as invasive carcinoma [46].

HER2 overexpression has been also documented in LG-IEN, although with significantly lower frequency (4–8%) compared to that found in HG-IEN (16–20%) [41–43]. On the whole, these findings suggest that HER2 overexpression characterizes the early steps of gastric carcinogenesis [41–43]. However, the absence of HER2 overexpression in invasive GC matching HER2-positive dysplasia indicates that this molecular deregulation may involve only a subset of cells in the intraepithelial neoplastic population [42].

By using immunohistochemistry, gastric dysplasia has been also classified into adenomatous/type I (intestinal phenotype), which is characterized by immunostaining for CD10 and CDX2; foveolar or pyloric/type II (gastric phenotype), which shows staining for MUC5AC and MUC6 and absence of CD10 expression; hybrid, which displays a mixed phenotype; null, when none of the aforementioned markers is expressed [47–50]. HER2 amplification was observed in cases classified as gastric or hybrid, which suggests that this type of dysplasia may represent the precursor of gastric type adenocarcinoma originating de novo from gastric mucosa [50]. An extensive analysis of HER2 status in immunoclassified gastric dysplasia may help to identify those patients at higher risk to develop a specific type of cancer, although the relationship between HER2 overexpression and progression of dysplasia to GC still requires further investigation.

## 3. HER2 in Early Gastric Cancer

There is some evidence that the identification of precursor lesions may be helpful for the early diagnosis of GC [51]. In Japan and Korea, endoscopy-based population screening allows frequent detection of early gastric cancer (EGC), which can be a suitable candidate for conservative treatments such as endoscopic submucosal dissection [51]. EGC is defined, irrespectively of the tumor size, as a carcinoma invading the mucosa and/or submucosa with or without lymph node metastases [52]. The incidence of nodal metastases in EGC depends upon the size, depth of invasion in the gastric wall, and histological differentiation of the tumor [53–55]. In detail, the incidence of nodal involvement is 0% for well-differentiated tumors of less than 2 cm in size and restricted to gastric mucosa, while it is higher than 30% for tumors showing infiltration in the submucosa, poor differentiation, and size larger than 2 cm [53–55].

According to the macroscopic classification of Japanese Endoscopic Society, EGC is divided into Type I, which includes tumors with polypoid growth, Type II which comprises tumors with superficial growth, Type III which describes tumors with excavating growth, and Type IV which refers to tumors with infiltrative growth and lateral spreading. Then, Type II EGC is further subdivided into IIa (elevated), IIb (flat), and IIc (depressed) and, on microscopic viewpoint, the most common histological architecture found in EGC is well differentiated, tubular, and/or papillary pattern [56]. For this reason, it may be challenging at times to discriminate between well-differentiated adenocarcinoma and high grade dysplasia, especially in superficial specimens of gastric mucosa [56]. EGC has good prognosis, with 5-year survival rate around 90% for N0 tumors [57] and around 70–75% for N+ carcinomas [57].

**Figure 2:**
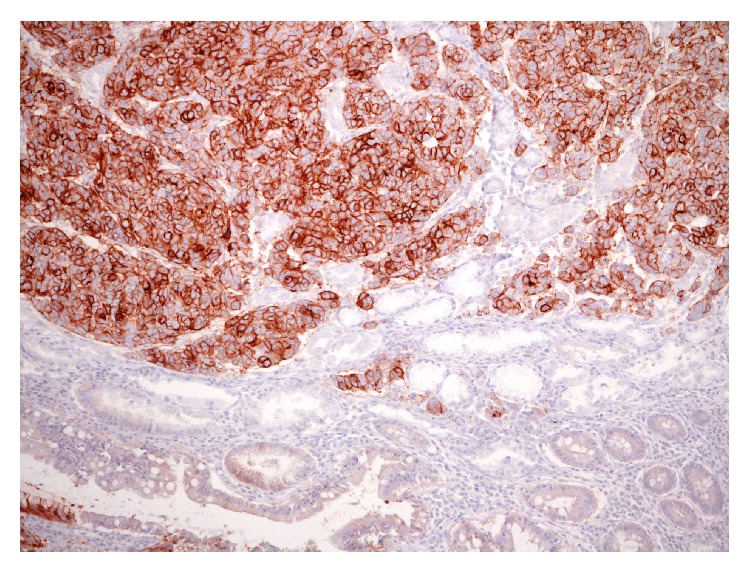
Intramucosal early gastric cancer with 3+ HER2 positivity; adjacent intestinal metaplasia present was unstained (original magnification, ×160; Mayer's Haemalum nuclear counterstain).

The presence of lymph node metastases is the main factor conditioning the surgical procedure for the resection of EGC. Indeed, according to the National Comprehensive Cancer Network guidelines [58], EGC without lymph node metastases can be a suitable candidate to endoscopic mucosal resection (EMR) or endoscopic submucosal dissection (ESD) [55, 59, 60]. The size, histological type, depth of invasion, and lymphatic or venous invasion of the primary tumor were evidenced as factors predictive of nodal metastases in EGC [61–65]. With reference to molecular alterations, microsatellite instability (MSI), mutations in the p53 gene and overexpression of the epidermal growth factor receptor (EGFR) and HER2 genes seem to have a prognostic role in EGC. In detail, high microsatellite instability (MSI-H), a form of genomic instability associated with defective DNA mismatch repair, was demonstrated in EGC with a frequency ranging between 8.2% and 37% [65–67] and it was shown as an independent predictor of low frequency of lymph node metastases and long survival in this subset of tumors [65, 68]. On the other hand, mutation in the* p53 *gene, which is one of the most frequent genetic abnormalities observed in GC, was associated with nodal metastases in EGC [65]. Finally, overexpression of EGFR and HER2 genes was significantly correlated with disease recurrence and poor prognosis in patients affected by EGC [65, 69, 70]. As a matter of fact, patients with HER2-negative pN0 EGC have significantly higher 5-year overall survival (91.1%) compared to patients with HER2-positive (Figure 2, authors' collection) pN0 EGC (81.8%) [60]. In addition, HER2 immunoexpression appears to be significantly associated with development of micrometastases in pN0 EGC [60, 71].

## 4. HER2 in Advanced Gastric Cancer

According to the published literature, HER2 overexpression/amplification, assessed by immunohistochemistry and/or in situ hybridization, ranges between 7% and 34% in advanced GC [72–78]. Of note, based on the results of an international randomized controlled trial (ToGA), patients with advanced gastric adenocarcinoma overexpressing HER2 are eligible for target treatment with Trastuzumab [22, 79]. Indeed a significant reduction of mortality rate was observed in patients with HER2 overexpressing advanced GC treated with combined chemotherapy and Trastuzumab [22, 79]. On the whole, HER2 positivity is significantly more frequent in gastroesophageal junction cancer (24–35%) compared to GC (9.5–21%) [73, 78, 80, 81]. Moreover, the rate of HER2 overexpression varies according to the histotype of GC [73, 75–77, 80], with higher frequency evidenced in the intestinal histotype (81.6%–91%) compared to the diffuse or mixed (4%–7.9%) [77, 82–85]. Of note, the pattern of HER2 immunoreactivity is frequently heterogeneous in intestinal GC, which showed intermingled HER2-positive and HER2-negative areas. On the other hand, a more uniform unreactive HER2 pattern was encountered in diffuse histotype. Interestingly, HER2 overexpression rate progressively increases moving from the poorly cohesive WHO histotype to the mitochondrion-rich adenocarcinoma (MRC), tubular adenocarcinoma, and hepatoid carcinoma (HAS) [74, 76] which has the highest frequency of HER2 positivity and the worst prognosis [74, 76]. HER2 overexpression is also significantly associated with high histological grade, high Ki-67 labeling index (LI), and advanced stage [78]; thus it represents an additional morphological parameter reflecting aggressiveness of GC [78]. The biological reasons for the peculiar association between HER2 overexpression and the histotype of GC have not been yet fully elucidated and additional investigation is required. However, a possible explanation for this phenomenon may reside in the relationship between e-cadherin and HER2 expression. Indeed HER2 amplification is inversely associated with e-cadherin mutations [75, 86], and e-cadherin mutations are frequent in diffuse gastric and lobular breast carcinoma and rare in intestinal and ductal breast cancer [73, 75].

HER2 overexpression/amplification is frequently heterogeneous in GC [46, 87, 88] compared to breast cancer, in which HER2 heterogeneity is uncommon [89, 90]. For this reason, several recommendations on methodology, interpretation, and quality control for HER2 testing in GC have been proposed, especially with regard to the assessment in bioptic specimens of surgically unresectable cases. In addition, criteria for the assessment of HER2 amplification in bioptic and surgical specimens of GC have been significantly modified from those routinely applied to breast carcinoma [91]. In particular, the guidelines for the assessment of HER2 status in GC state that the staining intensity (light, moderate, and strong) and distribution (complete, lateral, and basolateral) at cell membrane should be evaluated in at least 10% of neoplastic cells in surgical specimens and in a cluster of at least 5 tumor cells in the biopsy [77, 82, 87] (Table 1). This HER2 scoring system represents a reliable tool for the evaluation of HER2 status in GC biopsy and surgical specimen, and it results in good concordance between paired biopsy and surgical specimen of advanced GC, mainly if all the available specimens are tested [46, 77, 92–96]. Nonetheless, a low rate of HER2 discordance has been reported between paired biotic and surgical samples of GC [97].

No guidelines are currently available on the number of tumor blocks to be tested for HER2 expression. However it was proposed that more than one (at least three) representative tumor blocks, obtained from different neoplastic areas, should be analyzed in order to overcome HER2 heterogeneity [82, 92, 98]. Moreover, it was suggested that at least 6 to 8 tumor fragments are required for adequate assessment in biopsies, mainly in patients who have low chance of being submitted to surgery [46, 77].

**Figure 3:**
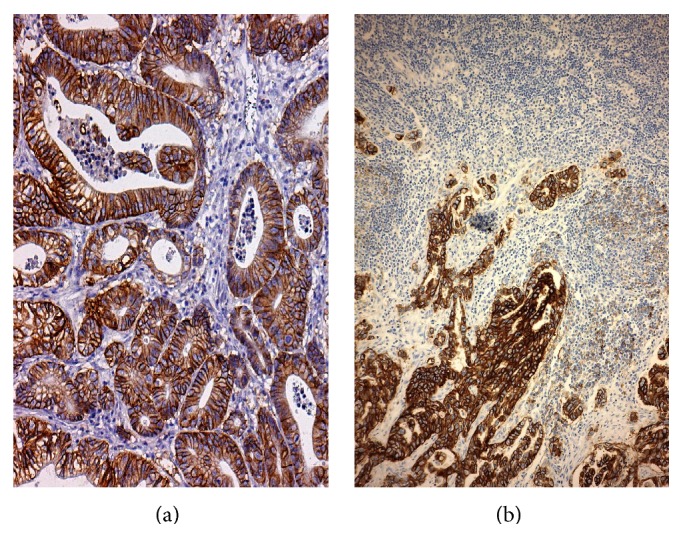
(a) Concordant HER2 status in primary GC (original magnification, ×320; Mayer's Haemalum nuclear counterstain) and (b) corresponding metastatic lymph node (original magnification, ×160; Mayer's Haemalum nuclear counterstain).

**Figure 4:**
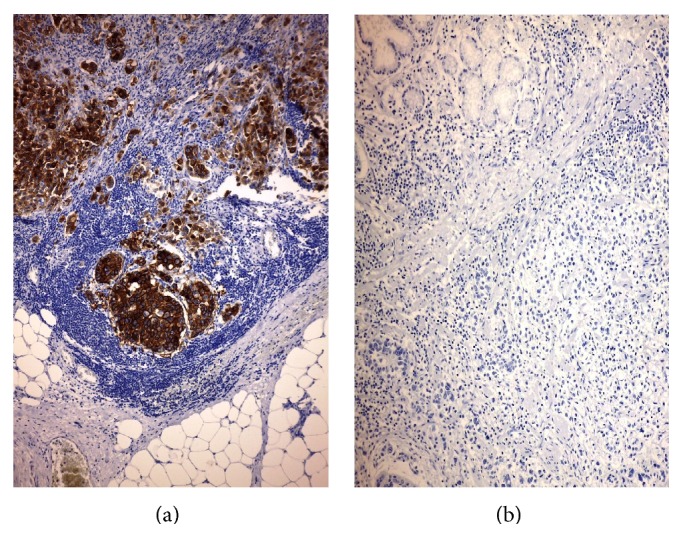
(a) Positive HER2 conversion in metastasis (original magnification, ×160; Mayer's Haemalum nuclear counterstain, ×160) in comparison to (b) negative primary GC (original magnification, ×120; Mayer's Haemalum nuclear counterstain).

Recently, several studies addressed the issue of HER2 concordance between primary carcinoma and its metastases (Figures 3 and 4, authors' collection). Indeed it was shown that HER2 status may differ between primary tumor and matched metastases in both breast and stomach cancers [99–108]. Although a preliminary report did not show any significant changes in HER2 status in metastatic lesions compared to primary GC [84], more recent data demonstrated discordant HER2 status between primary carcinoma and synchronous or metachronous locoregional/distant metastases, with a mean rate of 7% and either positive or negative conversion [41, 99, 101, 104, 109–113]. In addition, changes in HER2 status, consisting in either positive or negative conversion, were evidenced in a comparative analysis between paired primary GC and corresponding synchronous metastatic lymph nodes in patients who did not receive adjuvant chemotherapy [101, 104, 108]. This latter fining may have relevant clinical impact [108]. Indeed, if HER2 expression is tested only in the primary GC, a percentage of patients with HER2-positive conversion in lymph node metastases may be excluded from targeted therapy [108]. Positive conversion may be related to the development of a HER2-positive subclone in metastatic lymph nodes as a result of disease progression [108]. On the other hand, negative conversion observed in metastatic deposits of patients who had not received any neoadjuvant treatments [108] cannot develop as the result of resistance to Trastuzumab therapy. Of note, discrepancy in HER2 status between primary tumor and paired nodal metastases was already highlighted in breast cancer [106, 107]. Although at present there is no indication of testing HER2 status in synchronous nodal metastases from GC, possible discordance in HER2 expression in metastatic tumors compared to primitive cancer is relevant for the therapeutic management and prognosis of the patients. Indeed further patients eligible for Trastuzumab-based therapy may be identified by assessing HER2 status in synchronous metastases from patients with HER2-negative primary GC.

## 5. Conclusions

HER2 putative role in gastric carcinogenesis still needs investigation. The evidence of a higher rate of HER2 overexpression in gastric HG-IEN compared to LG-IEN suggests that HER2 may be involved in the early steps of gastric carcinogenesis. In accordance, GC showing CIN, frequent amplification of genes related to receptor tyrosine kinase RTK/RAS signaling such as HER2, and Lauren's intestinal type has been recognized as a distinct molecular subtype of GC [19, 114].

Although HER2 has emerged as a new therapeutic target in GC, its role as a prognostic marker in this tumor is still controversial [115–121]. Indeed, some studies demonstrated that HER2 overexpression is a poor prognostic factor in GC [122, 123], while others showed that it may be favorable or irrelevant for prognosis [85, 123, 124]. In view of the correlation between HER2 overexpression and the immunohistochemical subtype of gastric dysplasia, HER2 assessment in gastric dysplasia may be helpful in order to identify patients at increased risk of developing a specific type of cancer. In addition, in our opinion, HER2 testing can be used as a prognostic factor to predict the risk of poor outcome in EGC, since patients with HER2-negative pN0 EGC have significantly higher 5-year overall survival compared to patients with HER2-positive pN0 EGC [60].

In advanced GC, HER2 overexpression is significantly more frequent in tumors showing tubular histotype, high histological grade, advanced stage, and high Ki-67 LI, which suggests that it may represent an additional prognostic negative parameter. Finally, in view of the possible difference in HER2 status between primary GC and synchronous lymph node metastases, we suggest that HER2 status is routinely assessed not only in primary GC, but also in nodal and distant metastases, in order to identify possible candidates eligible for targeted Trastuzumab therapy.

## Figures and Tables

**Table 1:** Immunohistochemical criteria for HER2 scoring in neoplastic specimens of the stomach.

Surgery	Biopsy	HER2 score
No reactivity or membranous reactivity in <10% of tumor cells	No reactivity in any tumor cell	Negative (0)

Faint or barely detected membranous reactivity in ≥10% tumor cells	Tumor cell cluster of ≥5 cells with faint or barely detected membranous reactivity irrespective of percentage of tumor cells stained	Negative (1+)

Weak to moderate complete, basolateral, or lateral membranous reactivity in ≥10% tumor cells	Tumor cell cluster of ≥5 cells with weak to moderate complete, basolateral, or lateral membranous reactivity irrespective of percentage of tumor cells stained	Equivocal (2+)

Strong complete, basolateral, or lateral membranous reactivity in 10% or more of tumor cells	Tumor cell cluster of ≥5 cells with strong complete, basolateral, or lateral membranous reactivity irrespective of percentage of tumor cells stained	Positive (3+)
